# Application of next-generation sequencing technology to diagnosis and treatment of focal segmental glomerulosclerosis

**DOI:** 10.1007/s10157-017-1449-y

**Published:** 2017-07-27

**Authors:** Yutaka Harita

**Affiliations:** Department of Pediatrics, Graduate School of Medicine, The University of Tokyo, 7-3-1 Hongo, Bunkyo-ku, Tokyo, 113-8655 Japan

**Keywords:** Focal segmental glomerulosclerosis, Steroid-resistant nephrotic syndrome, Next-generation sequencing, Whole-exome analysis

## Abstract

A broad range of genetic and non-genetic factors can lead to kidney injury that manifests as focal segmental glomerulosclerosis (FSGS), which can be classified into primary (idiopathic) and secondary forms. Previous genetic approaches to familial or sporadic cases of FSGS or steroid-resistant nephrotic syndrome identified causal mutations in a subset of genes. Recently, next-generation sequencing (NGS) approaches are becoming a part of a standard assessment in medical genetics. Current knowledge of the comprehensive genomic information is changing the way we think about FSGS and draws attention not only to identification of novel causal genes, but also to potential roles for combinations of mutations in multiple genes, mutations with complex inheritance, and susceptibility genes with variable penetrance carrying relatively minor but significant effects. This review provides an update on recent advances in the genetic analysis of FSGS and highlights the potential as well as the new challenges of NGS for diagnosis and mechanism-based treatment of FSGS.

## Introduction

Focal segmental glomerulosclerosis (FSGS) is a group of clinicopathological entities sharing the common feature of glomerular lesion within a subset of glomeruli (i.e., focal) involving only a portion of the glomerular tuft (i.e., segmental) [1]. Clinically, FSGS is a common cause of steroid-resistant nephrotic syndrome (SRNS) with approximately 30–50% of adults with FSGS being unresponsive to steroid therapy, and exhibits a large overlap with clinical diagnosis of SRNS in children and adults.

A broad range of genetic and non-genetic factors can lead to kidney injury that manifests as FSGS, which can be broadly classified into primary (idiopathic) and secondary forms [1, 2] (Table 1). Typical primary FSGS, characterized by the presence of nephrotic syndrome with an observable FSGS lesion by light microscopy and widespread foot process effacement by electron microscopy (EM) [3, 4], presents with the highest rate of progression to end-stage renal disease. Secondary FSGS, commonly characterized by the absence of nephrotic syndrome and presence of segmental foot process effacement by EM [3], occurs as an adaptive structural–functional response to known etiologic causes including genetic defects, viral infections, drugs, toxins, and responses mediated by altered glomerular hemodynamics [2].

**Table 1:** Classification of FSGS

	Mechanism
Primary FSGS	Circulating factor(s) (yet unidentified)
Secondary	
Adaptive FSGS	Reduced renal mass or functioning glomerular number
Genetic FSGS	High-penetrant genetic mutations in genes crucial for podocyte function
APOL1 FSGS	Low-penetrant APOL1 variation
Infection/inflammation-associated FSGS	Direct effect on podocytes or glomerular components/cytokine?
Medication-associated FSGS	Direct effect of drugs on podocytes

In both primary and secondary FSGS, data from human and experimental studies indicate that podocyte injury, depletion (podocytopenia), and subsequent damage to parietal epithelial cells are pivotal events [5, 6] in the morphogenesis of characteristic segmental lesions.

While damage to podocytes underlies all forms of FSGS, the etiology of podocyte injury and pathogenesis of primary FSGS have posed a conundrum for decades. Several studies have revealed that circulating factors within patient plasma can cause podocyte damage in vitro [7–9]. One case of recovery from FSGS after retransplantation of an allograft that failed in the first recipient as the result of recurrent primary FSGS [10], yielded clinical evidence of one or more specific disease-causing circulating factors in primary FSGS patients. Indeed, FSGS recurs in about 30% of patients after renal transplantation, further emphasizing the role of a circulating factor. Despite vigorous investigation of several candidate factors, to date, no single molecule has been consistently identified as the causal pathogenic element in primary FSGS [11–13]. Further, there is a complete lack of information about the genetic background of primary FSGS.

In contrast, recent genetic approaches to patients with familial or hereditary FSGS identified primal causal mutations in a subset of genes. The aim of this review is to provide an update on recent advances in the genetic analysis of FSGS and highlight the potential of this translational approach for diagnosis and treatment of FSGS.

## Monogenic cause of FSGS/SRNS

The field of research into the genetic cause of nephrotic syndrome arose from the discovery of podocyte proteins that play crucial roles in glomerular filtration. The first gene identified was the nephrin gene (*NPHS1*), whose mutations cause Finnish-type congenital nephrotic syndrome (CNS) [14]. Since the discovery of *NPHS1* in 1998, the list of causative genes has grown rapidly (Table 2). Many of these genes were first identified by positional cloning or homozygosity mapping in families affected by CNS, SRNS or familial FSGS, and can roughly be divided into two clinical categories: early-onset recessive forms of FSGS/SRNS/CNS, and late-onset autosomal dominant forms of FSGS/SRNS.

**Table 2:** Genetic causes of FSGS

Isolated NS	Isolated or syndromic NS		Syndromic NS	
Autosomal recessive				
*NPHS1*	*CRB2*	(Ventriculomegaly)	*PDSS2*	(Encephalomyopathy)
*NPHS2*	*COQ2*	(Encephalopathy)	*MTTL1*	(MELAS syndrome)
*NPHS3*	*COQ6*	(Deafness)	*SCARB2*	(Epilepsy)
*CD2AP*	*ADCK4*	(Seizure)	*SMARCAL1*	(Schimke immunoosseous dysplasia)
*PTPRO*	*LAMB2*	(Pierson syndrome)	*ITGB4*	(Junctional epidermolysis bullosa), (pyloric atresia)
*MYOIE*	*COL4A3*	(Alport syndrome)	*CD151*	(Pretibial epidermolysis bullosa), (deafness)
*EMP2*	*COL4A4*	(Alport syndrome)	*ZMPSTE24*	(Mandibuloacral dysplasia)
*FAT1*	*CFH*	(aHUS/C3 Glomerulopathy)	*ITGA3*	(Interstitial lung disease), (epidermolysis bullosa)
*ARHGDIA*	*CUBN*	(Anemia)	*WDR73*	(Galloway–Mowat syndrome)
*KANK1*			*SGPL1*	(Hypogonadism), (adrenal insufficiency)
*KANK2*			*LMNA*	(Lipodystrophy)
*KANK4*				
*TTC21B*				
*NUP93*				
*NUP107*				
*NUP205*				
*XPOS*				
*MAGI2*				
Autosomal dominant				
*ACTN4*	*WT1*	(Wilms tumor), (hermaphroditism), (genital anomalies)	*MYH9*	(Epstein syndrome)
*ANLN*	*LMX1B*	(Nail–patella syndrome)		
*ARHGAP24*	*INF2*	(Charcot–Marie–Tooth disease)		
*TRPC6*	*PAX2*	(Renal coloboma syndrome)		
X-linked				
	*COL4A5*	(Alport syndrome)	*GLA*	(Fabry disease)

Note that genes can be divided both by mode of inheritance, and by presence or absence of extrarenal manifestations. Several genes have been identified to cause both isolated FSGS in which mutations are associated with manifestations only in kidney or syndromic FSGS in which mutations are also associated with extrarenal manifestations

Causative genes for the recessive type are mainly expressed in podocytes, where they are involved either directly or indirectly in organization of the slit diaphragm (SD) and actin cytoskeleton (Table 2). Nephrin, a membrane-spanning glycoprotein, is the major component of SD [14]. Podocin, encoded by *NPHS2*, is a lipid raft component of SD [15] that interacts with nephrin [16]. In addition to providing a structural framework for the filtration barrier, SD components such as nephrin and podocin also play important roles as a signaling platform [17]. Recent analyses have identified nuclear pore complex components to be essential for glomerular permselectivity, with biallelic mutations of nuclear proteins 93, 107 and 205 (*NUP93/107/205*) and exportin 5 (*XPO5*) causing SRNS through aberrant Smad signaling [18, 19]. Genetic causes of autosomal dominant forms of FSGS/SRNS include mutations in molecules associated with cytoskeletal organization of podocytes [actinin alpha 4 (*ACTN4*), inverted formin 2 (*INF2*), anillin (*ANLN*), and Rho GTPase Activating Protein 24 (*ARHGAP24)*, SD proteins [transient receptor potential cation channel subfamily C member 6 (*TRPC6*)], and transcription factors [Wilms tumor 1 (*WT1*) and LIM homeobox transcription factor 1 beta (*LMX1B*)].

Some genes capable of causing hereditary FSGS/SRNS may have roles in multiple cell types and often impose phenotypic effects in extrarenal systems. Thus, genes can also be divided clinically based on whether genetic forms are accompanied by extrarenal manifestations (Table 2). Indeed, a number of clinically meaningful extrarenal phenotypes exist, many of which are prominent and often diagnostic. Examples include microcoria in patients with laminin beta 2 (*LAMB2*) [20] mutations, coloboma in patients with paired box gene 2 (*PAX2*) [21] mutations, Denys–Drash syndrome (pseudohermaphroditism and Wilms tumor) or Frasier syndrome (streak gonads and pseudohermaphroditism) in patients with *WT1* mutations [22], and nail–patella syndrome in individuals with *LMX1B* mutations [23]. Other extrarenal phenotypes include mitochondrial cytopathies, and bone or neurological disorders.

Notably, recent reports revealed that in patients with a mutation of these pleiotropic genes, FSGS may be the only presenting manifestation. For example, specific mutations within the homeodomain of *LMX1B* have been shown to cause isolated nephropathy without nail, patellar or skeletal abnormalities [23]. Similarly, some missense mutations of *LAMB2* were observed in congenital and infantile steroid-resistant nephrotic syndromes without apparent eye anomaly [24]. Biallelic crumbs 2 (*CRB2*) mutations cause both isolated early-onset SRNS and a severe phenotype manifesting as congenital nephrotic syndrome, exhibiting renal microcysts complicated by marked cerebral ventriculomegaly, gray matter heterotopia, and elevated levels of maternal serum alpha-fetoprotein and amniotic fluid alpha-fetoprotein [25–27]. Although mechanisms underlying the organ specificity of these mutations remain largely unclear, diagnosis based on genetic cause will have clinical significance in treatment of nephropathy and potential undiagnosed extrarenal phenotypes.

## Benefits of gene identification in clinical settings

As with other clinical tests, it is crucial for clinicians to consider the needs and rationale of genetic testing for the care of each patient with FSGS. If correctly performed, genetic testing of FSGS patients can be beneficial in several ways.

First, identification of a mutation is important for genetic counseling, as determining the mode of inheritance can facilitate family counseling regarding future pregnancies. Second, mutations in some genes causing syndromic nephrotic syndrome would dictate further screening of extrarenal phenotypes. For example, upon identification of a mutation within the *WT1* gene, a clinician should investigate the gender genotype of females to exclude an XY genotype with pseudohermaphroditism, and the patient should be screened for development of a Wilms tumor or gonadoblastoma [28]. Third, renal prognoses can differ on the presence of genetic abnormalities. In children and young adults with FSGS/SRNS, patients with identified gene mutations demonstrate a higher likelihood to progress more quickly to end-stage renal failure [29]. The result of mutational analysis can be evaluated based on the clinical course of patients with the same mutation described in the literature if exists.

The results of genetic testing can also affect treatment of FSGS. Indeed, the discovery of a causative mutation in FSGS/SRNS genes strongly suggests that the nephrotic syndrome is steroid resistant. Regarding response to immunosuppressive therapy, there are arguments both for and against efficacy. Indeed, the mechanism of action by which cyclosporine A (CsA) is beneficial may be non-immune, as direct effects on podocytes have been demonstrated in vitro and in animal models [30]. However, efficacy of immunosuppressive therapy is significantly different in SRNS patients with or without mutations, as none [31] or only 3% [32] with genetic SRNS experienced complete remission. Although several hereditary SRNS cases have been reported to at least partially respond to CsA [33–37], caution should be warranted when considering administration of immunosuppressive drugs in these patients due to possible renal toxicity. Long-term effects of immunosuppression therapy in asymptomatic proteinuric patients with causative mutation have not been established.

The identification of a monogenic cause can even open new therapeutic possibilities in a limited but potentially significant number of patients. In patients with mutations in coenzyme Q_2_ (*COQ2*), *COQ6*, coenzyme Q_8B_ (*ADCK4*), or decaprenyl diphosphate synthase subunit 2 (*PDSS2*), genes involved in CoQ_10_ biosynthesis, early initiation of CoQ_10_ supplementation may be beneficial to reduce proteinuria and FSGS progression [38–41]. However, the efficacy of CoQ_10_ treatment needs to be evaluated in a larger number of patients with mutations in these genes. In patients with *CUBN* mutations, vitamin B12 treatment may improve megaloblastic anemia which can be unnoticed before molecular diagnosis.

Finally, in the setting of an FSGS patient undergoing kidney transplant, the likelihood of posttransplant recurrence is very low in patients with gene mutation [42, 43], possibly as the result of exclusion of primary FSGS, which is supposedly caused by circulating factor in plasma. However, this does not hold true for all cases. In patients with Fin-major/Fin-major mutations in *NPHS1* gene, the posttransplant recurrence rate is as high as 30% [44–46], mainly due to posttransplant production of anti-nephrin antibodies.

## Modality of genetic analysis by next-generation sequencing

Sanger sequencing has been the ‘gold standard’ in diagnostics as it has high specificity and sensitivity. However, diagnosis of genetic nephrotic syndrome, which can result from single or multiple mutations in more than 50 candidate genes, entails significant cost and effort using conventional methods.

Recent advances in the simultaneous sequencing of short DNA fragments have provided a revolutionary new approach for medical genetics by drastically decreasing the cost, improving the accuracy, and greatly increasing the speed of generating sequence data [47]. Major advantages of this method include higher yield of positive results arising from parallel and comprehensive analysis of all known and even unknown genes. Three representative next-generation sequencing (NGS) approaches for gene analysis have been applied in clinical settings: (1) targeted enrichment of a gene set (gene panel); (2) whole-exome sequencing (WES), and (3) whole-genome sequencing (WGS), in which the entire genome is sequenced without employing methods for sequence selection [48, 49] (Table 3).

**Table 3:** Characterization of NGS methods for identification of causative mutations and comparison with Sanger sequencing

	NGS			Conventional method
	WGS	WES	Targeted panel	Sanger sequencing
Target	Whole genome	Whole exonic regions	A set of targeted genes	Only one segment (−1 kb)
Description	Complete data set of an individual’s genome	Comprehensive assessment of exome	Succeed only if the causative gene is included in the panel	Gold standard test
Gene panel coverage	>97.5%	90–95%	−100%	−
Analysis of new disease genes	+	+	−	−
CNV calling	+ Calling of all structural variants possible	+ Calling of moderately large structural variants possible	+	−
Incidental finding	+	+	−	−
Intronic variants	+ (interpretation often challenging)	−	−	−
Cost and time	High cost (>3× of WES) and long analytic period	Considerably cheaper than WGS	Cheaper than WES	Cost- and labor-intensive to perform multiple genes
Sensitivity and specificity	Low	Low	High	High
Indication	Second line	First line	First line	Essential for confirmation of mutation

*CNV* copy number variation

While WGS is an unbiased approach for detecting genetic variations in both exons and introns, the inherent complexity of decoding the resulting massive data set (approximately 50-times larger than WES) and higher associated costs currently limit clinical utility in most settings. In WES, every exon of every protein-coding gene is enriched by one of several capture methods, sequenced by NGS, and then analyzed. As a focused and economical approach, WES is currently the more popular platform for discovery of rare-disease-causing genes. In gene panels, a system of amplification is used to isolate or enrich target regions. Panels can be optimized for coverage of target sequences, and consequently have higher read depth and accuracy compared with typical WES or WGS outputs, which improves the quality of sequencing. The other advantage of employing gene panels for clinical use is the reduced cost compared with WES or WGS. However, gene panels cannot reveal mutations in new genes not included within the panel, so panels must be updated regularly.

## Genetic analysis of FSGS/SRNS using NGS

Recently, several groups have used NGS to analyze the prevalence of genetic defects in large nephrotic syndrome cohorts. Representative reports are listed in Table 4. Some studies used WES, while others used gene panels analyzing from 20 to over 50 genes in a large number of individuals at a much lower cost per sample than traditional Sanger sequencing [50].

**Table 4:** Previous publications using NGS for diagnosis of FSGS/SRNS

Authors	Year	Journal	Modality	Number of genes	Number of patients	Gene detection rate (%)	References
McCarthy et al.	2013	*Clin J Am Soc Nephrol*	WES	24	36	19	[51]
Ding et al.	2014	*J Am Soc Nephrol*	WES	24	62	29	[78]
Lovric et al.	2014	*Clin J Am Soc Nephrol*	Gene panel	21	48	33	[50]
Giglio et al.	2015	*J Am Soc Nephrol*	Gene panel	19	31	32.3	[31]
Bullich et al.	2015	*Eur J Hum Genet*	Gene panel	26	25	36	[60]
Sadowski et al.	2015	*J Am Soc Nephrol*	Gene panel	27	1783	29.5	[52]
Buscher et al.	2016	*Clin J Am Soc Nephrol*	Gene panel	10	231	57	[32]
Gast et al.	2016	*Nephrol Dial Transplant*	Gene panel	39	81	20	[54]
Weber et al.	2016	*Pediatr Nephrol*	Gene panel	10	37	38	[59]
Bierzynska et al.	2017	*Kidney Int*	WES	53	187	26.2	[29]
Wang et al.	2017	*Pediatr Nephrol*	Gene panel	28	110	28.3	[55]

These investigations revealed that a high fraction of SRNS manifesting in childhood is caused by single-gene mutations. An earlier study analyzing 36 patients revealed that 70% of patients with familial cases and 15% of sporadic cases had a definitive or probable pathogenic variant identified [51]. The most comprehensive panel analysis of 27 known SRNS-causing genes in an international cohort detected a single-gene cause of disease in 29.5% of families [52].

However, there are several reasons why it is difficult to calculate true prevalence rates of gene abnormalities in nephrotic syndrome cohorts. First, with the exception of one recent study by a United Kingdom-based group that recruited SRNS patients as a national cohort in an unbiased manner [29], many studies are designed for discovery of variants rather than unbiased estimation of true effect sizes. Second, many papers present data on cohorts with overlapping patient groups. Often, they include patients in whom gene analysis for a certain gene has already been performed with a negative result, or those in whom mutations were identified by conventional Sanger sequencing prior to NGS analysis. Third, each research group used different statistical and functional criteria for assessing the causality of mutations, resulting in possible discrepancies between interpretations of each variant.

The most important and defined factor for prevalence of gene mutation in FSGS/SRNS is the age of patients, with the fraction of detecting single-gene causation being inversely correlated to age of manifestation. In the largest cohort, proportions of gene identification were 69.4, 49.7, 25.3, 17.8, and 10.8% in patients with disease manifesting during the first 3 months of life, 4–12 months, 1–6, 7–12, and 13–18 years, respectively [52, 53]. This study also revealed that causative genes differed significantly by age of manifestation. The most common causative genes identified within the first 3 months of life included *NPHS1, NPHS2, WT1* and *LAMB2*; whereas, *NPHS2* was the most frequent gene mutated in individuals with onset of SRNS between 1–16 years [52]. In an analysis of adult patients with FSGS/SRNS, Gast et al. found collagen (*COL4A3*–*5*) mutations to be most frequent, with identification in 38% of families with familial FSGS and 3% with sporadic FSGS [54]. Generally, mutations in dominant genes are rarely observed in early childhood, but more frequent in early adulthood.

Attention must also be given to ethnic groups within the cohort, as causative genes differ significantly by race. For example, in Chinese pediatric SRNS patients, *ADCK4* was the most commonly mutated gene, while only 3.33% of patients exhibited an *NPHS2* mutation [55]. Rarity of *NPHS2* mutations in Japanese pediatric patients with CNS or FSGS/SRNS has also been reported [56–58].

## Complexity of gene variations associated with FSGS/SRNS

As the success rate for detecting causal variants is far from complete, attention has also been given to potential roles for combinations of heterozygous mutations in multiple genes and complex or non-Mendelian inheritance.

In agreement with the substantial number of proteins required for properly functioning glomerular filtration, it is an attractive hypothesis that protein-altering variants within multiple known NS genes could lead to disease pathogenesis or be involved in disease severity. For example, NGS confronts us with the detection of secondary, possibly pathogenic variants. In the CNS/SRNS cohort with causative mutations identified for 38% of patients, a secondary variant within a different gene was also identified in 22% of patients [59]; although it is difficult to evaluate whether this finding is by chance or has any functional impact. Bullich et al. found two familial cases with mutations in both an FSGS/SRNS gene and *COL4A3* [60], and this combination of mutations was suspected to be related to increased disease severity. In contrast, a study applying rare variant association testing of 21 genes implicated in monogenic nephrotic syndrome for 393 patients in the Nephrotic Syndrome Study Network (NEPTUNE), led to the discovery that patients did not have a significantly increased burden of variants in Mendelian FSGS/SRNS genes compared with a reference cohort, nor was there any evidence for oligogenicity [61]. However, thus far, the possibility that rare variants insufficient to cause Mendelian disease can contribute to FSGS/SRNS as risk alleles and/or via oligogenicity cannot be dismissed.

Another example of complex inheritance is the p.R229Q *NPHS2* mutation. In contrast to typical early-onset SRNS by other *NPHS2* mutations, the p.R229Q mutation causes SRNS with a median age at diagnosis of 13 years and progression to ESRD by 26 years. Notably, homozygous p.R229Q mutation itself does not cause FSGS, but p.R229Q in conjunction with a additional disease-causing *NPHS2* mutation within 3′ regions is sufficient for late-onset SRNS [62, 63]. However, the pattern of inheritance is much more complicated, as the additional mutation alters heterodimerization and mislocalization of p.R229Q-encoded podocin, such that disease-associated 3′ mutations exert a dominant-negative effect on p.R229Q podocin, while it behaves as a recessive allele when associated with wild-type podocin. Therefore, the pathogenicity of a mutation can be dependent on other combined mutations within the gene or in other related genes.

## Inheritance of FSGS characterized by variable penetrance

Penetrance is defined as the percentage of individuals having a particular mutation or genotype who exhibit a phenotype related to the associated disorder or genotype. Complete penetrance indicates that all individuals who have the disease-causing mutation will exhibit clinical symptoms of the disease. With increasing amounts of extensive genomic data, questions regarding the incomplete penetrance of certain alleles for monogenic forms of FSGS/SRNS have also been unraveled. For instance, homozygous mutations in phospholipase C epsilon 1 (*PLCE1*, also known as *NPHS3*) were initially identified to cause a nonsyndromic, autosomal recessive form of diffuse mesangial sclerosis [33]. However, asymptomatic family members of affected children bearing the same homozygous mutation of *PLCE1* have also been reported [64, 65], indicating mutation of the *PLCE1* gene is not sufficient to cause diffuse mesangial sclerosis and, further, other modifier genes or environmental factors may play a role in the variability of renal phenotypes observed in individuals bearing *PLCE1* mutations. Variable disease penetrance in patients with mutations in *INF2* has also been reported. Within families with *INF2* mutations, there are rare individuals who harbor the variant, but remain clinically unaffected into their sixth and seventh decades of life [66]. Estimating penetrance is often difficult because it is sensitive to the clinical context and initial studies on index cases are only designed for discovery of variants but not for unbiased estimation of effect sizes. Thus, the true penetrance of variants in sporadically affected patients may be less than that estimated by initial publications based on key familial cases.

Genome-wide association studies (GWAS) have become a powerful approach to searching for common genetic variants capable of increasing susceptibility to complex diseases or traits. In contrast to Mendelian diseases, which have high penetrance and very rare allele frequency, alleles identified by GWAS demonstrate mild or modest effect sizes that cannot fully account for disease susceptibility. Thus far, a broader role for genetic susceptibility of both sporadic and familial cases of FSGS has been identified. The most significant genetic contributors to FSGS susceptibility are two genetic variants in apolipoprotein L1 (*APOL1*) [2, 67]. The effect is largely recessive, and the majority of individuals with two risk alleles do not exhibit renal disease. Despite low penetrance, the demonstration of kidney disease in transgenic mice expressing renal risk variants (but not the reference allele) [68] combined with the strength and consistency of genetic association is highly supportive of the causal role of *APOL1* genetic variants for FSGS. *APOL1*-associated FSGS is a major form of FSGS in sub-Saharan Africa, and approximately one-third of FSGS in the United States is associated with *APOL1* variants. Notably, *APOL1* risk alleles can be present in subjects who do not self-identify as having African ancestry [69]. Recent analysis identified other susceptibility genes that were validated to cause FSGS using mouse models [70].

## Difficulties for interpretation of NGS data

One of the most important steps of genetic diagnosis is to determine whether or not the variation definitively caused the disease. This is achieved by reliably separating genuine disease-causing or disease-associated genetic variants from the broader background of variants present in all human genomes. These variants may be rare and potentially functional, but may not actually be pathogenic [71]. If a patient is incorrectly informed that one of his or her variants is causal when in fact it is benign, the incorrect diagnosis causes adverse consequences to not only the patient, but also the patient’s entire family and possible descendants.

The American College of Medical Genetics and Genomics (ACMG) and Association for Molecular Pathology (AMP) developed an initial framework for interpretation of sequence variants as a consensus based on expert opinion, which has been widely used to classify variants as pathogenic or likely pathogenic [72]. In a case of a patient carrying one or more mutations identified in another family member as disease-causing, the judgment may be simple, although not decisive. It may be hard to decide the pathogenicity of a mutation that has never before been observed and which has not been studied biochemically or tested in an animal model. Information should be gathered about whether the mutation segregates with disease in a family, and the results of prediction software programs, such as SIFT and Polyphen, that evaluate likely pathogenicity of amino acid changes [73]. Regardless, these measures are not direct or perfect for assessing pathogenicity.

Since their development, population-level reference databases based on large human exome or genome sequencing such as ExAC or gnomAD [74] including Asian, African, Latino and other non-European ancestries have rapidly become a standard tool for medical genetics. Unfortunately, but inevitably, previous large-scale sequencing studies have falsely annotated common variants as disease-causing. A recent study revealed that about 10% of disease mutations in commonly used databases are incorrect, suggesting disease mutation annotations in such databases should be carefully scrutinized [75]. More recently, genetic variants common among African-Americans previously classified as disease-causing for hypertrophic cardiomyopathy were shown to be benign [76]. These misannotations stem from ascertainment bias and methodological shortcomings, such as excluding minority populations from control cohorts. Therefore, variant reclassification of previously published mutations, particularly for groups that have historically been understudied such as individuals of Asian or African ancestry, is urgent.

It should also be noted that, conversely, filtering out low-frequency variants may reduce sensitivity, as many recessive disease alleles (such as p.R229Q in *NPHS2*) are present at moderate frequencies in heterozygous or even homozygous states in population databases.

## Application of NGS for dissecting primary and secondary FSGS

As a causative humoral factor for primary FSGS has not been identified, there are no robust clinical indicators or biomarkers for primary FSGS. Moreover, prediction of disease progression or response to medication cannot be defined by plasma analysis. Identification of a causative gene mutation in an FSGS/SRNS patient implies that it results in structural defects of patient podocytes, and excludes the involvement of humoral factors. In contrast, it is unclear if the inability to identify a definitive mutation serves as an indirect measure to distinguish between primary and secondary FSGS.

Using morphometric analysis of podocyte foot processes in patients diagnosed with primary FSGS and FSGS secondary to maladaptive responses, Deegens et al. [77] observed differences in the degree of foot process effacement. In cases of primary FSGS, effacement was most severe, while foot processes were relatively preserved in secondary cases with little overlap between the two groups. Differences between clinical features, such as degree of proteinuria or partial steroid responsiveness, of primary and secondary FSGS have been proposed [3]. Over 90% of patients with initial steroid sensitivity who underwent kidney transplant had recurrence after transplantation, suggesting the primary form of FSGS [78]. However, as information about degree of foot process effacement, proteinuria, and response to steroid therapy were not included in the majority of previous studies employing NGS, it is unknown whether these clinical parameters have predictive value for mutation detection. Recent findings by a United Kingdom-based group revealed no mutations in SRNS patients with initial steroid sensitivity and secondary steroid resistance. Moreover, these patients demonstrated the highest risk of posttransplant recurrence [29], suggesting major involvement of an as yet identified circulating factor, but not genetic variation, in patients with initial steroid sensitivity. Detailed analysis based on documentation of clinical features and renal pathology may provide a means to further stratify FSGS on possible pathogenesis factors.

## Conclusion and perspectives

NGS has changed the landscape of FSGS, and nephrologists now have the ability to enable translation of NGS into diagnostic tools. Based on potential benefits of mutation identification include choice of appropriate therapy, awareness of subclinical extrarenal manifestation, estimation of prognosis (including posttransplant recurrence), and family issues, gene analysis using NGS will be recommended for the following FSGS/SRNS patients; (1) pediatric and young adults; (2) patients with extrarenal manifestation; (3) patients with family history of kidney disease or extrarenal manifestation, and (4) patients receiving kidney transplant. Future systematic studies based on stratification of patients by genetic information are needed to establish the application of NGS technologies to care for FSGS. Given the heterogenic nature of FSGS, the spread of NGS-based “bench to bedside” translational approaches will provide breakthroughs for its diagnosis and mechanism-based treatment.
